# 1H Nuclear Magnetic Resonance (NMR)-Based Metabolic Changes in Nucleus Accumbens and Medial Prefrontal Cortex Following Administration of Morphine in Mice

**DOI:** 10.7759/cureus.79972

**Published:** 2025-03-03

**Authors:** Ozra Dehkordi, Stephen Lin, Safia F Mohamud, Richard M Millis, Paul Wang

**Affiliations:** 1 Neurology, Howard University College of Medicine, Washington, USA; 2 Radiology, Howard University College of Medicine, Washington, USA; 3 Pathophysiology, American University of Antigua, St. Johns, ATG

**Keywords:** in-vivo 1h nmr, medial prefrontal cortex, mice, morphine, nucleus accumbens

## Abstract

Introduction: It is well known that opiate addiction is a neurobiological disease associated with dysregulation of multiple neurotransmitters and neurochemicals. Previous* ex-vivo* ^1^H nuclear magnetic resonance (NMR) studies have yielded mixed findings concerning opiate-induced neurometabolic changes at key reward-addiction sites. Whether such changes reflect the conditions in a live animal remains unknown. The present study was therefore designed to fill this knowledge gap by determining the effects of morphine-induced neurometabolic changes under in-vivo conditions.

Methods: In-vivo ^1^H NMR spectroscopy (SA Instruments, Stony Brook, NY) was used to measure neurochemical changes in nucleus accumbens (NAc) and medial prefrontal cortex (mPFC) of mice, subjected to twice-daily injections of morphine (10 mg kg^−1^ s.c.) for five days.

Results: Morphine induced significant changes in the concentrations of a number of metabolites in both mPFC and NAc. The glutamine component of the glutamine-glutamate-GABA excitatory-inhibitory cycle, increased in both mPFC and NAc. Significant increase in glutamate was also observed at mPFC, but not in NAc. The phosphocreatine, marker for energy metabolism, and the N-acetylaspartate marker for neuronal viability and energy metabolism decreased significantly in both mPFC and NAc. Glycerophosphocholine + phosphocholine, markers for cell membrane integrity, increased significantly in both NAc and mPFC after morphine. The antioxidant neurometabolites taurine and glutathione increased significantly in NAc; however, taurine decreased, and glutathione was unchanged in mPFC after morphine. Inositol, a marker for neuroinflammation, increased significantly in NAc.

Conclusion: The present study is the first in-vivo ^1^H NMR spectroscopy in mice to demonstrate morphine-induced dysregulation of multiple metabolites and neurochemicals within the reward-addiction neurocircuitry.

## Introduction

Morphine, one of the most effective opioid analgesics for management of acute or chronic pain, is known to produce tolerance, severe withdrawal symptoms and rewarding properties with a high risk of relapse [1,2]. Studies using genetic knockout mice have demonstrated that the analgesic, respiratory depressant and addictive effects of morphine and other clinically used opiates are mediated primarily through activation of μ-opioid receptors [1,3-5]. Neurons express μ-opioid receptors in several brain regions involved in the reward-addiction neurocircuitry: including ventral tegmental area (VTA), nucleus accumbens (NAc) and medial prefrontal cortex (mPFC) [6-8]. However, the precise neurometabolic changes associated with morphine-induced activation of μ-opioid receptors in the immediate milieu of these reward-addiction sites are not known.

The mechanism of opioid addiction involves dopaminergic modulation within these regions: activation of μ-opioid receptors inhibits GABAergic neurons in the VTA, leading to increased dopamine release in the NAc and other components of the mesolimbic system [9]. This surge in dopamine plays a central role in reinforcing drug-seeking behavior. Additionally, the mPFC, a key region in the reward-addiction circuitry, is crucial for decision-making, impulse control, self-regulation, emotional processing, and motivation - all of which are significantly disrupted by opioid addiction [10].

In addition to the well-known dopaminergic system, recent studies suggest that other neurotransmitters and neurometabolites may be implicated in the development of addiction to morphine. Several studies have reported a range of metabolic abnormalities in different brain regions associated with repeated morphine administration in animals and opioid-dependent humans. One of the techniques to evaluate such changes is high-resolution nuclear magnetic resonance (NMR) (SA Instruments, Stony Brook, NY) spectroscopy that can provide a spectral analysis of multiple neurotransmitters and endogenous metabolites near reward-addiction sites [11,12]. Ex-vivo ^1^H NMR spectroscopy-based studies have reported metabolic abnormalities induced by repeated morphine administration in different brain regions of rhesus monkeys [13] and rodents [14,15]. In-vivo ^1^H NMR spectroscopy in patients with prescription opioid dependence has also reported that glutamate (Glu) concentrations in the NAc of opioid addicts were significantly different from that of controls [16]. While these studies report changes in the concentrations of several neurometabolites including Glu, glutamine (Gln), and γ-aminobutyric acid (GABA), the specific changes varied depending on the species, brain region studied, duration of drug administration and ex-vivo versus in-vivo conditions. In the present study, we applied an in-vivo ^1^H NMR spectroscopy-based metabolomic approach to simultaneously measure changes in the concentrations of multiple neurotransmitters and metabolites in NAc and mPFC of mice subjected to repeated administration of morphine or saline. To our knowledge, this is the first in-vivo ^1^H NMR animal study to evaluate metabolic changes associated with morphine at reward-addiction sites.

## Materials and methods

Animals

All experiments were performed in CD-1 (M/F) mice (two to three months old) weighing 20-25 g. All animal procedures including the anesthesia and surgery were conducted in accordance with the guidance of the Institutional Animal Care and Use Committee (IACUC) of Howard University (Approval # IACUC-MED-21-02, October 2021-October 2023). All efforts were made to minimize the number of animals used and their suffering.

Approach

Male and female mice (N=16) weighing 20-25 g were used for this study. Animals were randomly assigned to two groups (n=8 animals per group) receiving morphine sulfate (10 mg kg^−1^ s.c.) or saline (0.2 mL, s.c.) twice a day for five days. This dosage of morphine is known to cause morphine dependence [17] and morphine-conditioned place preference [18]. Morphine was dissolved in saline and was injected in a volume of 0.2 mL/injection. To reduce the nonspecific effects of handling and experimental environment, all animals were handled and exposed to the same environment for a total of 3 days. On day 4, animals were anesthetized (isoflurane in 0.8L/min oxygen at 2%-3% for induction, 1%-2% for maintenance) and underwent baseline (pre-exposure) ^1^H NMR spectroscopy. On days 5-9, the mice received morphine twice a day. On day 9, after the last injection, the animals were anesthetized and underwent a second round of ^1^H NMR spectroscopy.

^1^H NMR spectroscopy

An animal monitoring unit (SA Instruments, Stony Brook, NY) was used to monitor respiration during imaging and magnetic resonance spectroscopy acquisition. Depth of anesthesia was assessed throughout the study by monitoring the respiration rate, with target respiration range 40-50/min during scanning. A 9.4T Bruker AVANCE 89mm bore MRI machine (Bruker, Billerica, MA) was used with a 25mm RF volume coil. A set of T1-weighted (TE=8.4ms, TR=800ms) and T2-weighted (TE=33ms, TR=2500ms) spin echo pilot images were acquired in an orientation matching a mouse stereotactic brain atlas [19] to identify key landmarks for positioning the region of interest (ROI) for spectroscopy. A fieldmap-based localized shimming was applied over the ROI followed by iterative first order shimming, nominally achieving <15Hz for water peak. Localized ^1^H NMR was acquired with a point resolved spectroscopy (PRESS) sequence (TE=15ms, TR=2.5s, 1,024 averages) with variable power and optimized relaxation (VAPOR) delays for water suppression from each of the NAc and mPFC regions. The order of ROI acquisition was balanced across the mice. The ROI size for NAc was 2.0×2.0×2.0 mm^3.^ The ROI for mPFC was 2.0×2.0×1.0 mm^3^. The ROIs were identified from a mouse brain atlas and matched with the MRI images. The ROI (bregma 0.97-1.69 mm) of NAc in the axial view was placed in the left hemisphere using the following morphology: the caudal edge of the ROI was aligned with the first appearance of the anterior part of the anterior commissure. The ROI was centered 0.2 mm lateral to the midline and its ventral edge coincided with the ventral edge of the brain. The ROI of mPFC (bregma 1.33-2.33 mm) in the axial view was centered in the midline and located medial to the lateral ventricles, dorsal to the NAc. The center of the ROI was aligned with the corpus callosum.

Data processing and statistical analysis

Given the well-documented biological, hormonal, and genetic differences in opioid metabolism between males and females, our study incorporated both sexes to ensure comprehensive and generalizable findings. To minimize sex-related variability, we employed a within-subject design, where each mouse served as its own control. By directly comparing each animal's response to treatment with its own baseline measurements, we minimized the impact of sex-related changes in metabolites. All the NMR spectra were analyzed using LCModel software (LCMODEL Inc., Oakville, ON) [20] to obtain the absolute concentrations of neurotransmitters and metabolites in NAc and mPFC. LCModel uses a linear combination of model spectra acquired from in-vitro solutions to fit the acquired spectra. A basis set of these model spectra matching the field strength and acquisition parameters of the study (TE=15ms), was generated and provided by Dr. Provencher of LCModel Inc. This basis set includes L-alanine (Ala), aspartate (Asp), creatine (Cr), phosphocreatine (PCr), γ-aminobutyric acid (GABA), glucose (Glc), glutamine (Gln), Glu, glycerophosphocholine (GPC), phosphocholine (PCh), glutathione (Gsh), myo-inositol (Ins), lactate (Lac), N-acetylaspartate (NAA), N-acetylaspartylglutamate (NAAG), scyllo-inositol (Scyllo), and taurine (Tau), along with simulated lipids and macromolecule compounds. Using an unsuppressed reference water signal, LCModel provides metabolite concentrations along with their associated Cramér-Rao Lower Bounds (CRLB), expressed as percentages (%SD). The CRLB represents the uncertainty in the concentration estimate, with lower values indicating more reliable quantification. Metabolite concentrations with a %SD below 20% were considered reliable and included in statistical analyses, while higher %SD values indicate greater uncertainty. For example, the CRLB values for Glu in the mPFC ranged from 4.35% to 3.8%, and the CRLB values for Gln ranged from 9.9% to 12.33%. LCModel calculated the weighted mean and standard deviation (SD) of these metabolite concentrations by factoring in their CRLB-derived standard deviations. All data were expressed as weighted mean ± SD. A two-tailed paired t-test was used to compare metabolite concentrations before and after repeated subcutaneous administration of saline or morphine. P ≤ 0.05 was considered significant. The changes in the weighted concentrations of metabolites between day 0 and day 5 were calculated for both saline and morphine treated animals. Significance of the saline and morphine treatment-related differences was determined by two-tailed t-test for independent samples using the standard error of the mean (SEM). SEM was computed from the SD of the weighted mean concentrations before and after treatment.

## Results

**Figure 1:**
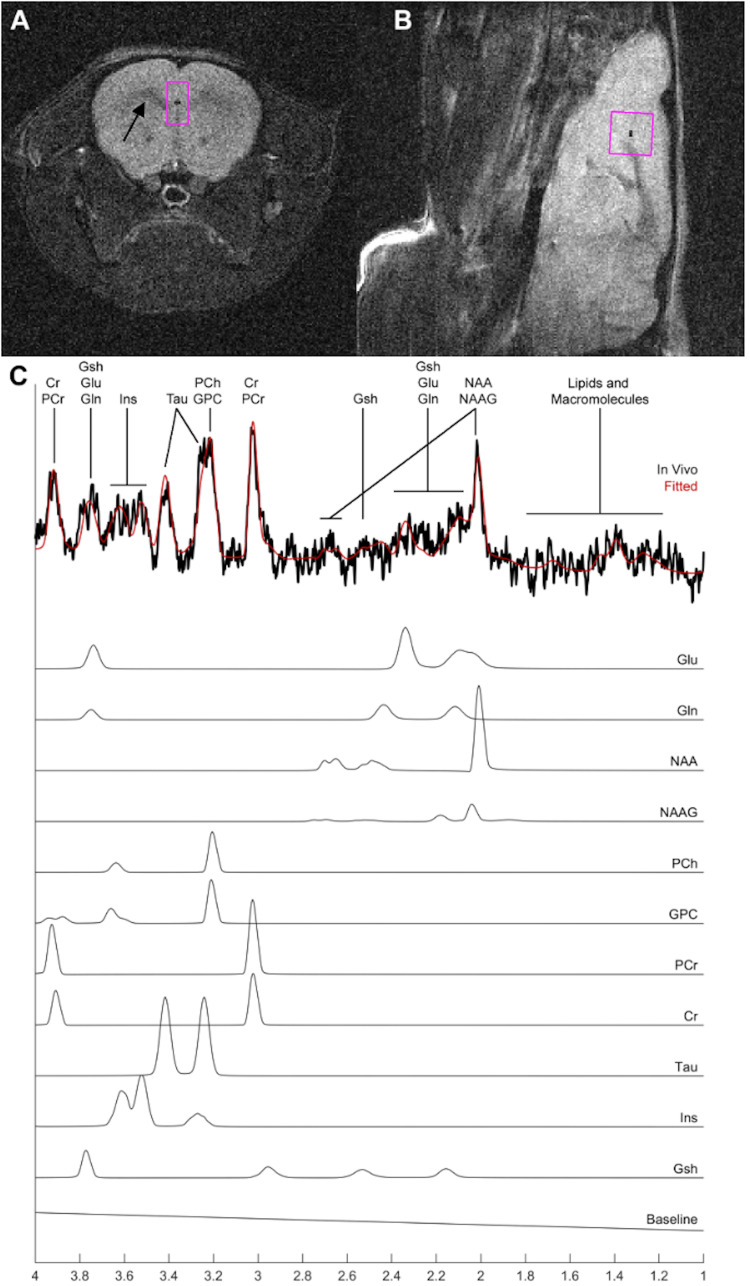
1H nuclear magnetic resonance spectroscopy at medial prefrontal cortex. Representative in vivo ^1^H NMR spectroscopy study of the medial prefrontal cortex (mPFC) of a control mouse. (A, B) Axial and sagittal pilot images, respectively, highlighting the mPFC region of interest (size 2.0×2.0×1.0 mm^3^). (C) Typical spectrum from the mPFC region of interest in (A, B) with fitted spectrum from LCModel (red) and subspectra of neurological metabolites such as glutamate (Glu), glutamine (Gln), N-acetylaspartate (NAA), N-acetylaspartylglutamine (NAAG), phosphocholine (PCh), Glycerophosphocholine (GPC), phosphocreatine (PCr), creatine (Cr), taurine (Tau), inositol (Ins), and glutathione (Gsh) that can be reliably identified in naïve animals. LCModel uses the subspectra to quantify the absolute concentration of each metabolite. Arrow: Corpus Callosum

**Figure 2:**
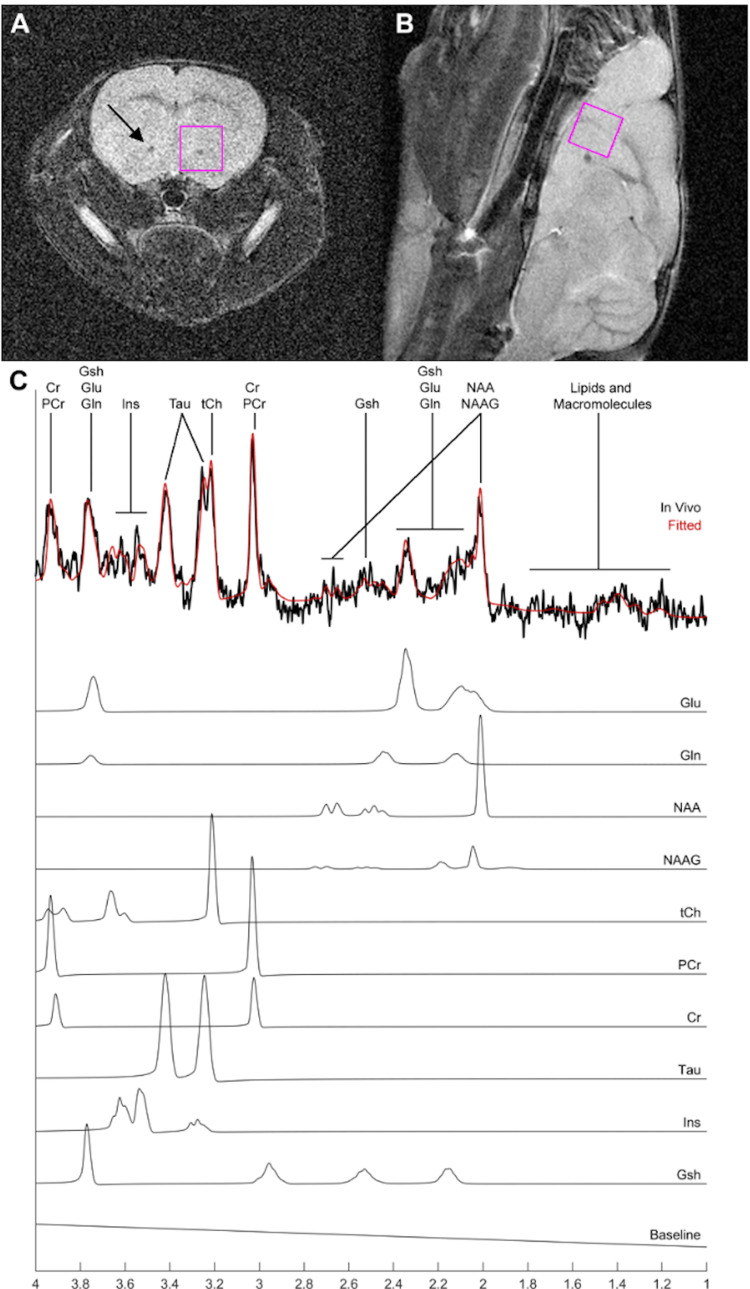
1H magnetic resonance spectroscopy at nucleus accumbens. Representative in vivo ^1^H NMR spectroscopy study of the nucleus accumbens (NAc) of a control mouse. (A, B) Axial and sagittal pilot images, respectively, highlighting the NAc region of interest (size 2.0×2.0×2.0 mm^3^). (C) Typical spectrum from the NAc region of interest in (A, B) with fitted spectrum from LCModel (red) and subspectra of neurological metabolites such as glutamate (Glu), glutamine (Gln), N-acetylaspartate (NAA), N-acetylaspartylglutamine (NAAG), phosphocholine (PCh), Glycerophosphocholine (GPC), phosphocreatine (PCr), creatine (Cr), taurine (Tau), inositol (Ins) and glutathione (Gsh) that can be reliably identified in naïve animals. LCModel uses the subspectra to quantify the absolute concentration of each metabolite. Arrow: Anterior commissure (anterior part)

Figures 1A-1C, 2A-2C show representatives in-vivo ^1^H NMR spectroscopy recordings at mPFC and NAc obtained from a control mouse. Neurometabolic profiles composed of multiple neurotransmitters and various metabolites and their combinations were quantified. The neurotransmitters and metabolites which met the criteria for inclusion were as follows: Cr, PCr, Gln, Glu, Gsh, Ins, NAA, Tau, GPC+PCh, NAA+NAAG, Cr+PCr, and Gln+Glu.

Metabolite changes after saline treatment

**Figure 3:**
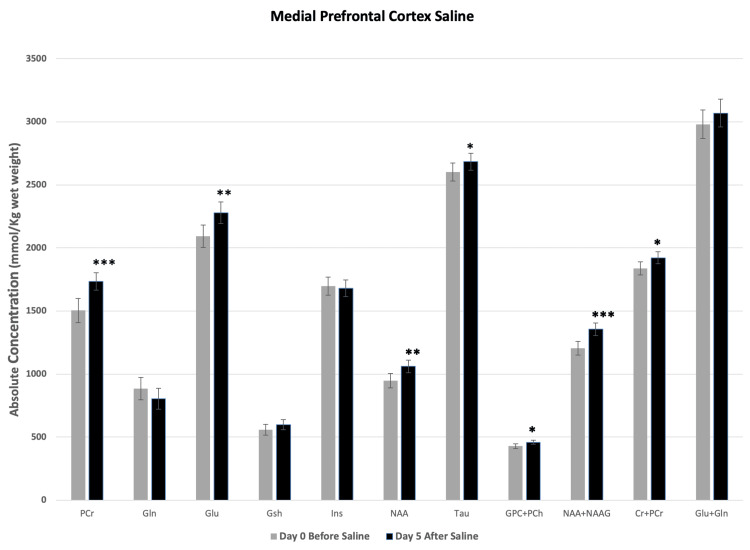
Saline-induced changes in metabolites at medial prefrontal cortex (mPFC). Metabolite concentrations quantified from mPFC of mice before and five days after twice a day subcutaneous administration of saline. Two-tailed t-test was used to compare metabolite concentrations before and after saline. Data represent mean ± SD, N=8. Significance level: *P < 0.05, **P ≤ 0.001, ***P ≤ 0.0001.

**Figure 4:**
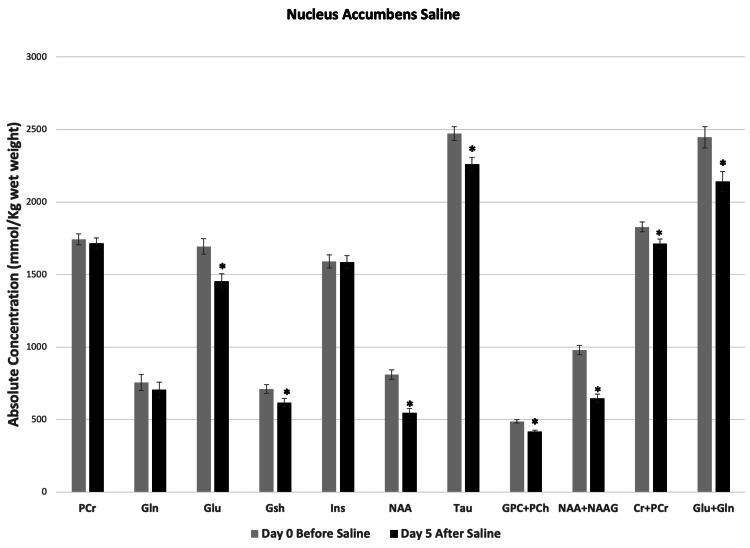
Saline-induced changes in metabolites at nucleus accumbens (NAc). Metabolite concentrations quantified from NAc of mice before and five days after twice a day subcutaneous administration of saline. Two-tailed t-test was used to compare metabolite concentrations before and after saline. Data represent mean ± SD, N=8. Significance level: *P < 0.0001.

Saline administration induced significant region-specific changes in the concentrations of a number of metabolites in both mPFC and NAc. In mPFC, concentrations of PCr, Glu, NAA, NAA+NAAG, GPC+PCh and Cr+PCr increased significantly (Figure 3). However, in NAc, except PCr, Gln, and Ins, all the other metabolites decreased significantly after saline injection (Figure 4).

Metabolite changes after morphine treatment

**Figure 5:**
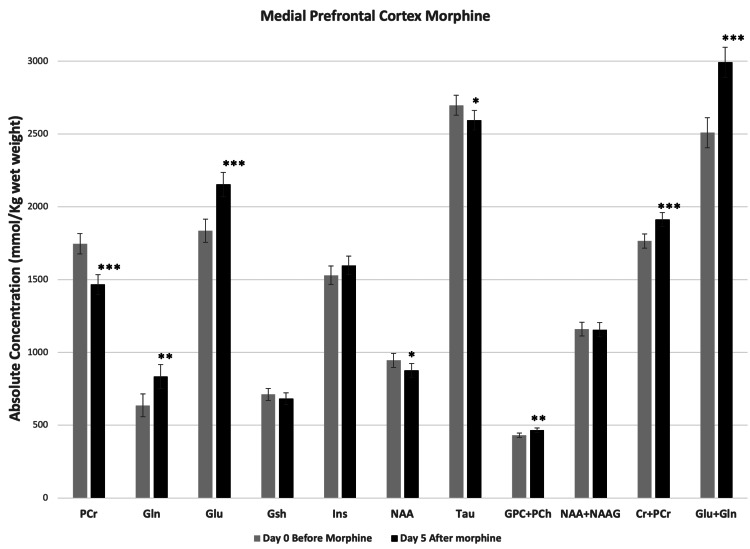
Morphine-induced changes in metabolites at medial prefrontal cortex (mPFC). Metabolite concentrations quantified from mPFC of mice before and five days after twice a day subcutaneous administration of morphine. Two-tailed t-test was used to compare metabolite concentrations before and after morphine. Data represent mean ± SD, N=8. Significance level: *P < 0.05, **P < 0.001, ***P < 0.0001.

**Figure 6:**
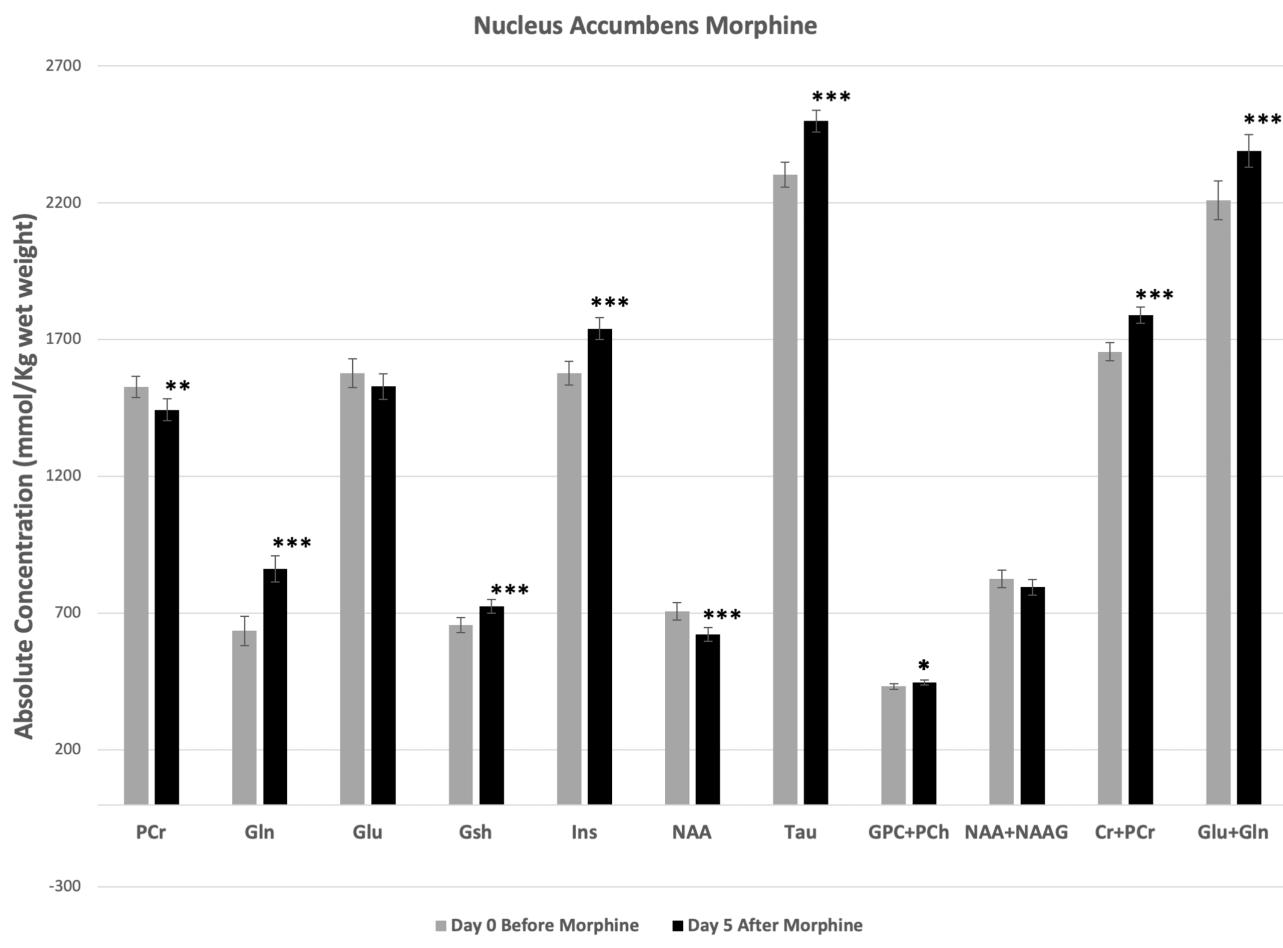
Morphine-induced changes in metabolites at nucleus accumbens (NAc). Metabolite concentrations quantified from NAc of mice before and five days after twice a day subcutaneous administration of morphine. Two-tailed t-test was used to compare metabolite concentrations before and after morphine. Data represent mean ± SD, N=8. Significance level: *P < 0.05, **P < 0.001, ***P ≤ 0.0001.

Figures 5, 6 show the neurochemical changes in mPFC and NAc, respectively, associated with the repeated subcutaneous administration of morphine. Gln+Glu increased significantly in both NAc and mPFC. The Gln+Glu increase in mPFC was due to significant increases in both Gln and Glu; whereas the increase in Gln+Glu in NAc resulted from a significant increase in Gln alone. The metabolites involved in energy metabolism also changed significantly after repeated morphine administration. PCr decreased in both mPFC and NAc, whereas Cr+PCr increased significantly in both regions. NAA, a marker of neuronal viability and energy metabolism, also decreased in both mPFC and NAc; however, NAA+NAAG did not change. The antioxidant markers Gsh and Tau both increased in NAc, but in mPFC Tau decreased whereas the Gsh change was not significant. Morphine treatment did not change the level of Ins, a neuroinflammatory marker, in mPFC; however, the level of Ins increased in NAc. GPC+PCh, markers for cell membrane integrity, increased significantly in both NAc and mPFC after morphine.

Morphine vs. saline treatment

**Figure 7:**
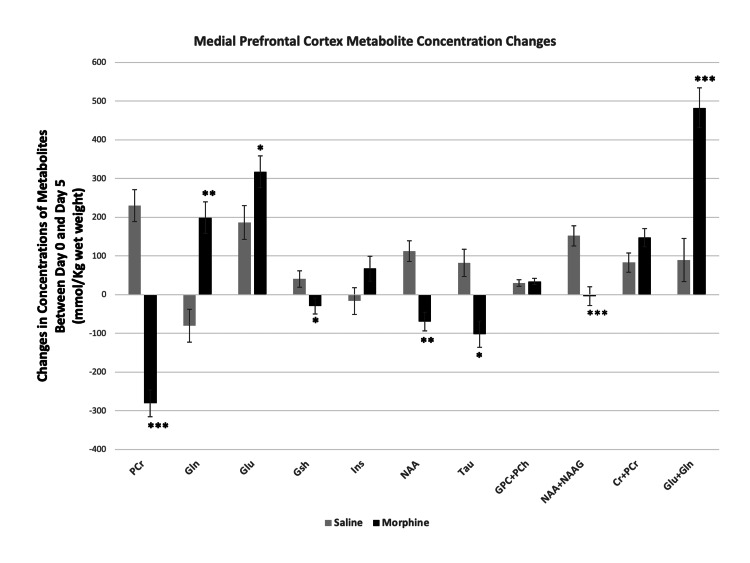
Comparison of saline and morphine-induced metabolite changes at medial prefrontal cortex. Changes in the concentrations of metabolites between day 0 (no treatment) and day 5 of saline and/or morphine treated mice. Significance of the saline and morphine treatment-related differences was determined by two-tailed t-test for independent samples. Data represent mean concentration differences for each metabolite (day 0 - day 5) ± SEM. Significance level: *P < 0.05, **P < 0.001, ***P ≤ 0.0001.

**Figure 8:**
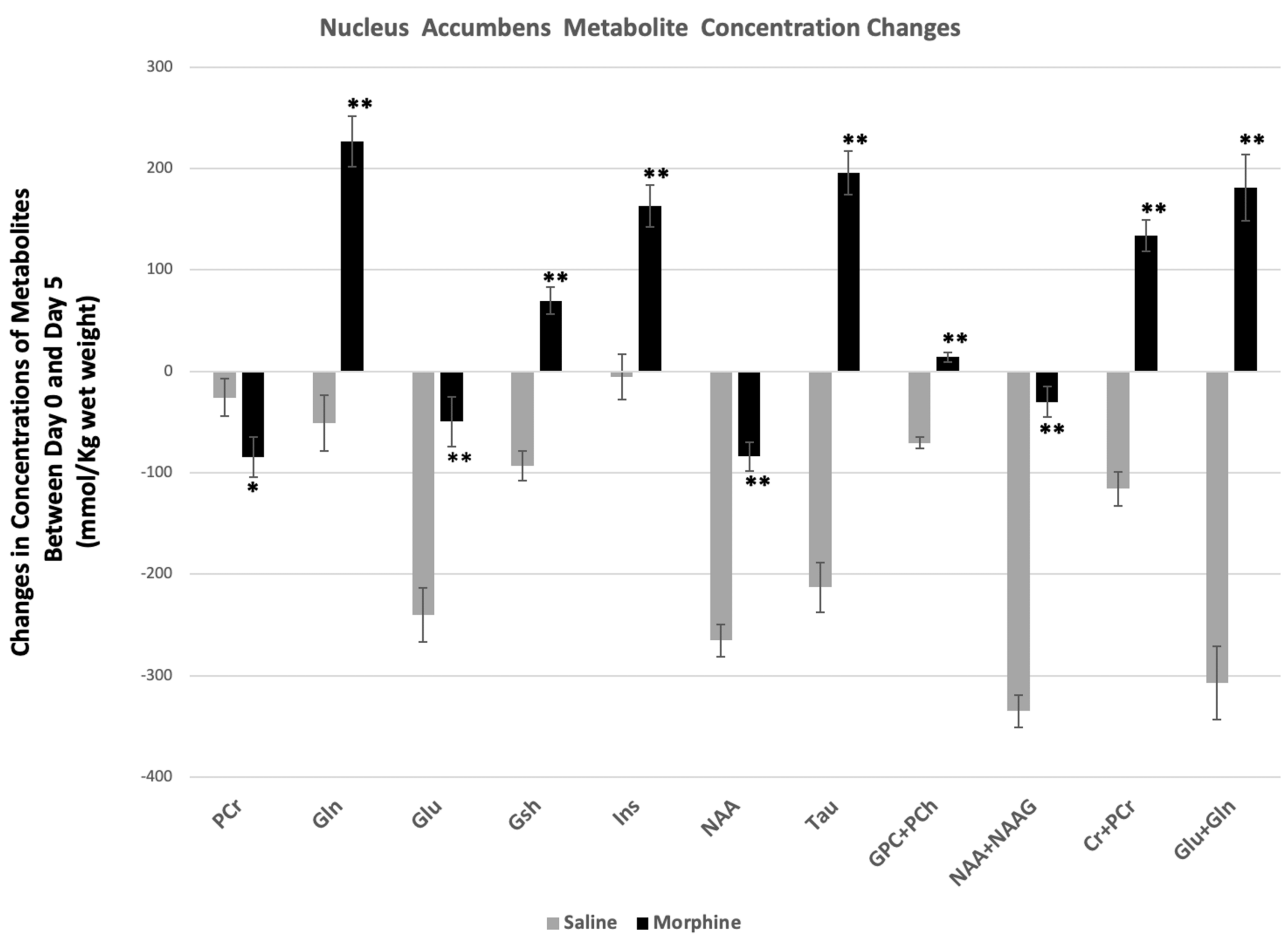
Comparison of saline and morphine-induced metabolite changes at nucleus accumbens. Changes in the concentrations of metabolites between day 0 (no treatment) and day 5 of saline and/or morphine treated mice. Significance of the saline and morphine treatment-related differences was determined by two-tailed t-test for independent samples. Data represent mean concentration differences for each metabolite (day 0 - day 5) ± SEM. Significance level: *P < 0.001, **P < 0.0001.

Comparison of the changes in concentrations of metabolites between day 0 and day 5 in saline and morphine treated animals showed significant differences between the two groups. PCr, Gsh, NAA, NAA+NAAG and Tau increased in mPFC after the saline treatment, whereas these same metabolites decreased after the morphine treatment. The direction of changes in other metabolites including Glu, and Glu+Gln in mPFC was the same in both the saline and the morphine-treated groups, but the magnitude of changes was significantly different between the two groups (Figure 7). In NAc, there was also a significant difference between the saline and morphine treated animals concerning either the direction and/or the magnitude of changes in metabolites between day 0 and day 5. The concentrations of PCr, Glu, NAA, NAA+NAAG decreased in both the saline and the morphine-treated groups, but the magnitude of changes was significantly different between the two groups. All other metabolites at NAc increased from their control after morphine treatment, whereas these same metabolites decreased after saline treatment (Figure 8).

## Discussion

The present in-vivo ^1^H NMR spectroscopy study in mice demonstrates that repeated subcutaneous administration of saline or morphine produces significant region-specific changes in several neurotransmitters and various metabolites at both NAc and mPFC. The saline group received subcutaneous administration of a minute amount of physiological isotonic sodium chloride, unlikely to produce brain effects attributable to the sodium chloride content per se. Therefore, the observed metabolic alterations in this group are more likely a consequence of experimental stress associated with repeated injections. In vivo ^1^H NMR studies in both human and animals have reported stress-related alterations in metabolite levels across various brain regions [21,22]. However, discrepancies exist regarding the direction of these metabolic changes, particularly in key metabolites such as Glu and those involved in energy metabolism. These variations are thought to depend on factors such as stress duration, stress type, and the specific brain regions examined. For instance, acute stress has been shown to elevate Glu levels in mPFC, whereas chronic stress leads to a reduction in Glu within this region [23,24]. Furthermore, as demonstrated in the present study, experimental stressors such as handling and saline injections induce distinct metabolic effects across different brain regions, including the mPFC and NAc, highlighting the complexity of stress-induced neurochemical responses. In contrast, morphine and other opioids are known to attenuate stress-related behaviors [25-27]. Thus, the metabolic changes observed following repeated morphine administration are likely driven by its anti-stress effects and its well-established role in addiction-related neuroadaptations.

In the present study, Gln+Glu increased in both mPFC and NAc following the morphine treatment. In mPFC, this increase in Gln+Glu was accompanied by a simultaneous increase in both Gln and Glu, implying morphine-induced enhancement of the Glu excitatory neurotransmission marker at mPFC. In NAc, the increase in Gln+Glu was associated with an increase in Gln and no change in Glu. Gln is a known precursor for Glu, Asp, and GABA [28]. Since the present experimental setup did not permit quantification of Asp and GABA, the significance of this increase in Gln on these excitatory-inhibitory neurotransmission markers at NAc remains unclear.

Several studies have reported morphine-induced disturbances in the Gln-Glu-GABA cycle. Animal and human data regarding the specific effects of morphine and other opioids on these metabolites at NAc and mPFC are inconsistent. In-vivo microdialysis and in-vitro studies have shown that morphine suppresses both basal and evoked increases in extracellular Glu in NAc and other brain regions [29-31]. Ex-vivo ^1^H NMR studies in the rat have reported an increase in Gln and Glu in NAc, but a decrease in both Gln and Glu in mPFC following prolonged administration of morphine [15]. Other ex-vivo ^1^H NMR studies in the rat have reported decreases in Glu with no significant effects on Gln in PFC following repeated morphine treatment [14]. In the present study, the observed increase in Gln+Glu levels in mPFC and NAc is consistent with human findings of elevated Gln+Glu in the anterior cingulate cortex (ACC) of long-term opioid users [32]. Likewise, in agreement with our findings in the mPFC, Glu levels in the ACC have been reported to be higher in heroin-dependent individuals receiving low versus high doses of methadone [33]. However, variability across studies may result from differences in experimental conditions, including in-vivo versus ex-vivo methodologies, duration of opioid exposure, and imaging techniques. Despite these discrepancies, both preclinical and clinical evidence suggest that morphine disrupts the Gln-Glu-GABA cycle [14,15,29-31], underscoring the translational significance of rodent models in studying opioid-induced neurochemical dysregulation.

In the present study, PCr decreased, whereas Cr+PCr increased in both mPFC and NAc after morphine treatment. Morphine inhibits the activity of creatine kinase B (CKB), an enzyme known to catalyze the reversible phosphorylation of creatine by ATP to produce PCr and ADP [34]. Thus, the decrease in PCr seen here may be due to morphine’s inhibition of CKB. PCr, a high-energy phosphate compound and the most immediate reserve for the re-phosphorylation of ATP, is known to play a vital role in cellular energy buffering and energy transport [35,36]. Thus, decreased concentration of PCr in both mPFC and NAc, may imply morphine-induced depletion of this energy reserve.

NAA, one of the most concentrated neuromodulators in the brain, was also found to decrease in both mPFC and NAc following repeated morphine administration. These in-vivo ^1^H NMR findings in mice are different than those reported for ex-vivo ^1^H NMR studies in morphine-dependent rats where significant increases in NAA were demonstrated at NAc, hippocampus, and striatum [15]. The significance of morphine-induced changes in NAA at reward-addiction sites is not known. NAA is believed to be present only in neuronal cell bodies and their axons [37-39]. The depletion of NAA in different CNS disorders such as ischemic stroke [40], multiple sclerosis [41], and Alzheimer disease [42] is thought to be a reflection of neuronal loss or dysfunction [39]. NAA appears to have several functions in the CNS including osmoregulation and energy metabolism [43-45]. Several studies have demonstrated decreases in NAA levels when brain energy metabolism was impaired by a variety of mechanisms, including brain injury. In traumatic brain injury, the decrements in NAA consistently correlate with reductions in ATP, implying that these reductions in NAA are related to energetic impairment [46-48]. LC-MS studies in humans have shown that NAA synthesis is directly coupled to glucose metabolism [49]. Thus, the decrease in NAA seen in the present study is most likely associated with a morphine-induced neuronal loss and/or dysfunction, as well as an impairment of energy metabolism.

NAAG, a highly concentrated dipeptide in the brain, is typically measured in conjunction with NAA in ^1^H MRS due to significant spectral overlap. NAAG contributes approximately 10%-20% of the signal commonly attributed to NAA [50,51]. Although structurally similar to NAA, NAAG functions as an inhibitory neurotransmitter, playing a crucial role in glutamate regulation and offering neuroprotection against excitotoxicity [52]. The observed decrease in NAA and the lack of a significant change in the combined NAA + NAAG signal after morphine may indicate a compensatory increase in NAAG levels. The underlying mechanism for this potential increase, whether due to glutamatergic regulation in mPFC or NAc, or a result of morphine’s effects on neuronal activity, remains unclear and warrants further investigation.

Tau, a sulfur-containing amino acid, increased in NAc and decreased in mPFC following repeated morphine administration. Tau has been reported to have multiple functions in the CNS including osmylyte, neurotransmitter, neuromodulator, and antioxidant [53]. Tau release from astrocytes has been shown to increase in response to hypo-osmolality, hypoxia, ischemia, metabolic toxins and oxidative stress [53-55]. Tau also acts as a free radical scavenger that can prevent damage from oxidative stress induced by toxicants [56,57]. Morphine use has been correlated with CNS oxidative stress and free radical production [58]. Thus, in the present study, the increase in the concentration of Tau at NAc is likely to be a protective response to prevent morphine-induced oxidative stress.

Gsh, an antioxidant metabolite, known to provide a major line of defense against oxidative stress, increased in NAc following morphine, but the changes in mPFC were not significant. ^1^H NMR-based metabolic studies in the rat have also reported changes in Gsh in NAc, PFC and striatum following chronic administration of morphine [15].

Ins, one of the most abundant metabolites in the human brain, also increased in NAc following morphine. Ins is mainly located in glial cells and is considered a marker for glial cell activity, proliferation and neuroinflammation [59,60]. Behavioral studies in rats have reported that glial cell activation and elevation of Ins in NAc and ventral hippocampus may represent a biomarker for addiction vulnerability [61]. Ins is reported to be higher in the brains of addicted patients than in healthy controls [62,63]. Morphine and other addictive drugs are known to cause cognitive impairments, which negatively correlate with Ins levels in humans and rodents [64-66]. The significance of elevated Ins in NAc is not known. However, recent studies in NAc have shown that, in addition to its role in behavior such as motivation, reward, and locomotion, NAc is also involved in learning and memory processes [67,68]. Further studies are needed to determine whether morphine-induced cognitive impairment is partially mediated by elevated levels of Ins in NAc. 

GPC+PCh, the two main choline-containing compounds in the brain, also increased in both mPFC and NAc following repeated administration of morphine. GPC+PCh are involved in cell membrane synthesis and degradation and are purported to be biomarkers for cell membrane integrity [69]. The implications of these changes following morphine are unknown; however, an increase in GPC+PCh implies that the oxidative and other metabolic stresses associated with morphine may have affected membrane integrity at these brain sites.

Limitations

The signal-to-noise ratio (SNR) of the acquired ^1^H NMR spectrum was a major concern when designing the study, given the small size and shape of the target brain regions. Thus, the size of the voxel (volume of interest) was selected to optimize the SNR, to maximize the coverage of target brain regions (mPFC and NAc) and to minimize contributions of extraneous tissues. However, the possibility that tissue from neighboring brain regions could contribute a small portion of the metabolic signal cannot be eliminated. The SNR associated with small voxel size also makes identification and separation of particular signals much more difficult. As a result, concentrations of metabolites with overlapping spectra are summated and reported as total. This evaluates morphine-induced changes of individual metabolites more challenging. Another limitation of the study is that we did not evaluate the effects of short versus long term administration of morphine and morphine withdrawal. While both sexes were included in the study, gender-based differences were not examined in the statistical analysis. Behavioral assessments of morphine dependence were not conducted. Therefore, our findings represent neurochemical and metabolic changes without direct behavioral correlations. However, previous studies in a different mouse strain have demonstrated that the dose used induces morphine dependence and conditioned place preference [17,18]. The use of isoflurane anesthesia also posed a limitation, as it significantly alters cerebral metabolism and blood flow, potentially introducing biases in the NMR-based metabolite measurements compared to awake animals [70]. To minimize this effect, baseline metabolite levels were measured after anesthesia induction but before morphine administration, thereby reducing variability and allowing for a more reliable evaluation of morphine-induced metabolic changes.

## Conclusions

In the present study, we used in-vivo ^1^H NMR spectroscopy in mice to measure the neurometabolic changes at NAc and mPFC associated with repeated administration of morphine. The differences between these in-vivo and previously reported ^1^H NMR studies may be explained by various factors, including in-vivo versus ex-vivo conditions, the animal species, durations, and dosages of morphine. The results suggest that disturbances in Gln and Glu seen in both mPFC and NAc are likely the consequence of morphine-induced changes in the excitatory-inhibitory balance associated with the Gln-Glu-GABA cycle. Changes associated with PCr and NAA in NAc and mPFC are likely due to morphine-induced disturbances in energy metabolism and neuronal dysfunction or integrity. Changes in GPC+PCh suggest an effect of morphine on cell membrane integrity. Altered levels of the antioxidant metabolites Gsh and Tau are likely a compensatory response to morphine-induced oxidative stress. Future clinical studies should validate these biomarkers in human opioid users and to assess whether the metabolic disturbances observed in active opioid use persist, reverse, or worsen during withdrawal and recovery. Such studies could help optimize detoxification protocols and inform strategies to prevent relapse.
